# ‘Let's work together to pass medical school’: a qualitative study of medical student attitudes to teamwork, competition and collaboration

**DOI:** 10.1002/2211-5463.13915

**Published:** 2024-11-07

**Authors:** Helen R. Watson, Paul Millin, James Close, Robert Jeffery, Holly Stephenson, Daniel Zahra

**Affiliations:** ^1^ Peninsula Medical School University of Plymouth UK

**Keywords:** assessment, employability, medical education, qualitative study, teamwork, transferable skills

## Abstract

Teamwork is vital to all types of work, and graduates of higher education programmes must be prepared to contribute to a wide variety of professional teams. This is especially true in healthcare, where graduates will work in multidisciplinary teams (MDTs) under considerable pressure. This study is a follow‐up to a previous study, where we described how competition between students is a barrier to constructive teamwork. Since then, we have made considerable enhancements to our transferable skills curriculum, moved away from norm referencing, and there have been national changes to the way that graduate Foundation training places are allocated. Here we present findings from a qualitative study of students from all six stages of our medical degree programme (5 years plus predegree foundation year). We explored whether there had been changes in how students perceived the importance of teamwork, their own teamwork development and how they collaborated with their peers. Following analysis of in‐depth, semi‐structured interviews, five themes emerged: (a) competition between students; (b) importance of teamwork; (c) what makes effective teamwork; (d) preparing for work in MDTs; and (e) recommendations for teamwork education. Competition between students was perceived as both positive and negative, but there has been a shift since our last study towards collaboration, with students now more willing to help each other succeed. Students also show more insight into their teamwork development, and were able to discuss what aspects of the programme, and higher education more broadly, were most valuable in helping them develop.

## Graphical abstract

Teamwork is vital to all types of work, and graduates of higher education programmes must be prepared to contribute to a wide variety of professional teams. Here we present findings from a qualitative study of students from all stages of a UK medical degree programme. The key themes that emerged are summarised here.

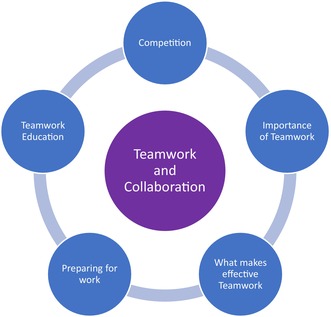

**Abbreviations**
BMBS: Bachelor of Medicine, Bachelor of Surgery
DaE: Doctor as Educator
EBL: enquiry‐based learning
IPE: interprofessional education
MDT: multidisciplinary team
NHS: National Health Service
SJT: situational judgement test
SSC: student‐selected component

Effective teams are key to almost all areas of work. Students graduating from our universities are likely to be employed by organisations that require them to work in teams of varied shapes and sizes, and it is vital that we prepare them to do this well [1, 2]. The need for our students to become valued team members transcends academic discipline or eventual field of work. Whilst this paper discusses teamwork development in UK medical students, the key findings are relevant across healthcare, science and other areas of higher education. Teamwork is one of several transferable skills that students need to develop regardless of their subject of study and whether or not they remain in that field when they enter employment.

Two years ago, we published our findings from a qualitative study, entitled ‘“Everyone is trying to outcompete each other”: a qualitative study of medical student attitudes to a novel peer‐assessed undergraduate teamwork module’ [3]. Whilst students appeared to benefit from this module taken in their fourth year of study, they noted competition between students being a hinderance to their teamwork development. Some progress was made in their development and understanding of teamwork, in terms of seeing the importance of it to their future clinical careers and gaining insight into their own areas of strength and weakness, but we felt there was greater scope to help students genuinely appreciate the importance of teamwork in healthcare and to provide more opportunities for them to develop and apply these vital skills [3]. A number of changes have occurred since this study, both internally within our medical curriculum and externally in the sector, including to the process by which medical students are allocated to postgraduate Foundation training places. This new study seeks to explore whether these changes have had any impact on our students' perception of teamwork development, how they reflect on their teamwork skills gained to date and how they relate this to their future careers. Because of the number of changes, and in order to expand on our previous findings, we have carried out an extended study, across all five stages of our undergraduate Bachelor of Medicine, Bachelor of Surgery (BMBS) and students on our pre‐BMBS foundation programme.

## Teamwork in medical education and higher education

**Fig. 1:**
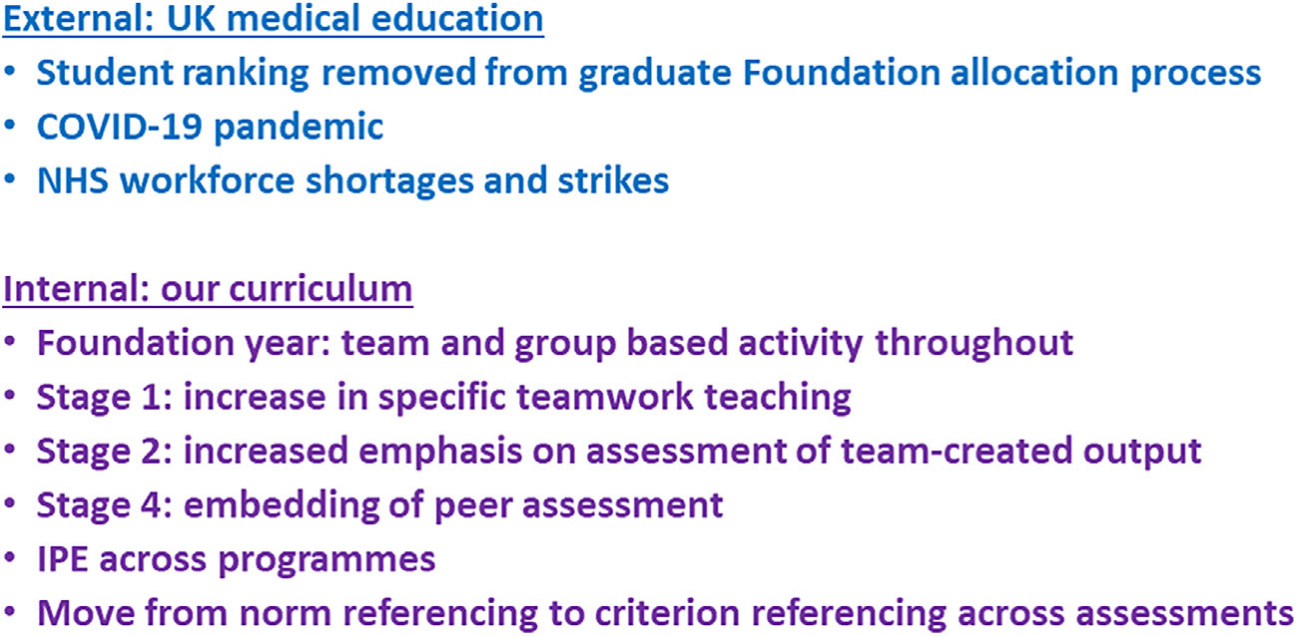
Summary of changes to medical education in the UK and to our programme. Key national and local changes discussed in this study are summarised here.

Teamwork and other key transferable skills are vital to the effective functioning of any organisation [1, 2]. Although these skills are generally assumed to be acquired during a university education, they are not routinely taught and assessed with the same emphasis as factual knowledge and subject‐specific skills. The General Medical Council and World Health Organization both specify the need for medical training to include teaching of teamwork skills, emphasising their importance in patient safety and good clinical outcomes [4, 5]. The UK Quality Assurance Agency specifies teamwork and other transferable skills as key outcomes from degree level qualifications, for example in the recently revised Biosciences Subject Benchmark Statement [6]. Yet there is a general consensus in the literature that we need to embed teamwork much more into our teaching and assessment in all educational contexts [7, 8]. Many higher education curricula include small group learning, team projects, and other nonlecture‐based teaching modalities, but it cannot be assumed that by teaching subject content in different contexts, students will automatically develop these skills and become competent [9, 10]. It is difficult to ‘teach’ and assess teamwork in a conventional way, as we cannot simply tell students about it in a lecture, ask them to read around the topic and then test them in an examination. Indeed, it has been noted in the literature by both academics and students themselves that currently used teaching and assessment methodologies only go a small way towards the development of teamwork that is required [11, 12]. A key factor in this is the concept that what students are assessed on will, to some extent, drive how they behave and what they spend time learning [13, 14]. In this context, without reliable and valid assessment tools, there is little emphasis on teamwork assessment, meaning that students may well focus their efforts elsewhere. We have been working in recent years to increase explicit focus on teamwork in our medical curriculum, including assessing various aspects of teamwork and work produced by teams of students. The specific details of this are covered below, to give some context to this study. Key changes and events in medical education in the UK, and changes to our medical programme, are summarised in Fig. 1.

## Changes in medical education and higher education

In the UK, graduates from medical degrees are placed into a two‐year Foundation Programme, to further expand their skills and knowledge before they select an area of medicine in which to specialise. Until 2024, Foundation places were allocated according to a ranking provided by the medical school (based on a student's attainment on their programme) and a nationally administered Situational Judgement Test (SJT). From the 2024 allocation round onwards, the SJT is no longer required, and medical schools are no longer asked to provide rankings for their students. Students are now allocated according to a ‘preference informed allocation process’ [15]. The advantages and disadvantages of this new approach will not be discussed here, but the change is highly relevant when we consider collaboration and teamwork in medical education and the ‘hidden curriculum’ it informs. The change means that for all current medical students, their ranking within their cohort will no longer feed into the Foundation allocation process. For students in the later stages of their programmes, the ranking system will have been present for a significant part of their training to date, so for them this represents a potential change of mindset, unlike our foundation students and students currently in earlier stages. We are therefore interested to see whether this reduction in direct and explicit competition between students has had any impact on their desire to work collaboratively and to contribute to shared team outputs. These changes were only implemented in the 2023/24 academic year, but students and staff were made aware of these changes prior to the start of the academic year. We are not aware of any studies yet published that seek to answer this question directly.

There are various other external factors which have undoubtedly influenced our students, including the COVID‐19 pandemic (where students were impacted variably, depending on stage of study) and ongoing strikes and workforce issues in the UK National Health Service (NHS). Whilst we have not investigated these issues specifically, the clinical experiences of our students occur within an extremely stretched healthcare system, where individuals and teams are under a huge amount of pressure [16].

Whilst the Foundation allocation process is specific to medical education, workload increases and other pressures are common across sectors, including in academia. Preparing our students to be part of effective teams, in the face of other pressures, is a key responsibility that we share across disciplines in higher education. Direct competition between students is present in many higher education settings too, for example in competition for prizes and awards, and where academic attainment informs allocation to prestigious placement experiences or years in industry.

## A teamwork curriculum: changes in our medical programme

Our previous study described the evaluation of a novel teamwork component introduced into the fourth year of our undergraduate BMBS medical degree programme, as part of the student‐selected component (SSC) module [3]. This was part of a wider review of transferable skills education, and several other changes have been made to our programme as a result. These changes are summarised below, alongside more recent changes made since this earlier work.

Our foundation degree is one route into medicine, with a significant proportion of medical entrants now completing this course prior to starting our BMBS programme. The foundation degree curriculum contains a number of team and group‐based sessions including enquiry‐based learning (EBL) and communication skills. Foundation students also have team‐based assessments. Whilst this has been the case for several years, we have included foundation degree students in our interviews this time, to gain their perspective on early teamwork training.

The SSC module is where much of our transferable skills education resides. Changes have been made to this module recently to increase the focus on teamwork. SSC in the first year includes some introductory teamwork education. Lectures are given to highlight transferable skills development, and students work in small groups (10 students) on their individual written assignments, some of which have been published [17]. There is an expectation that students help each other by giving and receiving feedback on their topic of choice. They are assessed by their group facilitator (a lecturer) with a professionalism judgement that includes some aspects of teamwork such as self‐awareness, communication and quality of feedback. We have more recently expanded the teamwork element into the SSC module in year two of the programme in our ‘Doctor as Educator’ (DaE) component. This is the first explicit team‐based assessment in the main medical programme, where students work together on a longitudinal project to produce a teaching resource and deliver a conference‐style presentation. They are assessed as a team, so all team members receive the same mark for the work they do together. This is the first time that they are reliant on others in their team to complete a piece of assessed work. They are also given a professionalism judgement which includes aspects of teamwork that build on the first year module. Continuing development of DaE has been guided by a staff‐student cocreation team where meaningful peer assessment has been identified as an important part of team assessment. The SSC in the fourth year has evolved somewhat since the last study, but still includes assessment of work produced as a team, and peer assessment. In this module, students have explicit formal teaching on teamwork, including using personality profiles [18], Belbin's team roles [19] and Tuckman's model of team development [20]. Across the years in SSC, students are given additional teaching on factors influencing team effectiveness, providing a theoretical background to the teamwork they practice during their programme. Emphasis in these sessions is on psychological safety [21], dependability, clarity of purpose and role and the concept of collective intelligence [22].

In addition to these specific programme changes, interprofessional education (IPE) has been prioritised across our Faculty, with more opportunities for our students to attend sessions with students from many other healthcare professions. For example, we have arranged regular sessions for BMBS students to work alongside Physician Associate students in EBL. IPE is certainly more visible to students in our School and Faculty than it was during our previous study.

A final set of changes that we asked students about in the interviews relates to a shift from norm referencing towards criterion referencing that is being implemented across our programme, that is shifting from grades that are relative to the performance of peers, towards grades fixed against marking criteria. The rationale for this change is to emphasise the achievement of expected standards over outperforming peers, where historically medical education had reflected the norms of direct competition in the sector that were until recently also enshrined in systems such as Foundation training placement rankings [23, 24]. Both the implicit and explicit expectations of students are major determinants of student behaviour, and assessment criteria and grading systems are a clear indication of what these explicit expectations are in higher education institutions [25, 26, 27]. As a result, these expectations have potentially important implications for student (and staff) attitudes to collaboration [3]. In this new system, where performance is described relative to an external, criterion‐based standard, all students can potentially achieve the top grade in any given assessment; something that is not possible with norm referencing methods where, by definition, a proportion will always fall into the tails of the score distribution and receive the associated grades, regardless of how high, or low, their absolute scores are.

Given the number of changes within our programme, our institution and sector, we have here sought to again explore our students' perceptions of teamwork, collaboration and competition. We aimed to gain an in‐depth understanding of how these changes have impacted our students and to find out more about their insight into teamwork, both in terms of their own skills development and how they see its application in their future careers.

## Methods

This study uses a similar approach to our initial study [3] but has been expanded to include a wider range of students and to explore how significant internal and external changes may have impacted student perceptions of teamwork and collaboration. We have again used an exploratory approach, aligning with social constructionist theories of learning [28]. The emphasis here is not to apply any one theoretical perspective, rather to explore our students' experiences in depth, and describe their opinions in detail.

This study was granted ethical approval by the University of Plymouth Faculty of Health Research Ethics and Integrity Committee, project ID 4691.

We interviewed 15 students from the BMBS programme, recruiting from all stages; two students from foundation (of 41 enrolled), three stage one (of 164 enrolled), two stage two (of 149 enrolled), three stage three (of 191 enrolled), two stage four (of 188 enrolled) and three stage five (of 150 enrolled). Interviews were carried out during February and early March of the 2023/24 academic year. The questions that guided these interviews were based on the previous study [3] with some modifications to reflect internal and external changes impacting collaboration and teamwork:

1. How important is teamwork to you? Why? (professionally and personally)
2. What do you think makes good teamwork? (*if they mention knowledge/theory at any point, then explore this further*) And what do you think might be barriers to good teamwork?
3. What areas of teamwork do you think you're good at?
4. What areas of teamwork do you think you could be better at?
5. Do you think your understanding of teamworking has changed during your medical degree so far? What's changed. What drove the change.
6. What specific experiences on the BMBS programme have facilitated your development of teamwork skills.
7. Rankings have been removed since early 2023—has this made a difference to how you work with your peers? If yes, can you tell me some examples of what you do differently now?
8. Do you feel any different since the rankings have gone? How?
9. Within the School we're also removing norm referencing (comparing you to your peers), moving towards a model whereby everyone could get Excellent—has this already, or do you think it would, impact how you work with your peers?
10. Given that multi‐disciplinary teams are the primary means of delivering healthcare, how are you preparing yourself for contributing to this as an F1 and beyond?
11. Do you think we should keep any elements of competition in the medical course, if so what?
12. What do you think our school could do to encourage more & better teamwork or cooperation between students?

Participants were recruited through an email that was sent to all students on all stages of the BMBS. Those who participated were compensated with a £20 voucher. Participation was entirely voluntary, and we recognise that the 15 students that were interviewed may not be representative of the entire student cohort. Before the interviews, all students were issued with a participation information sheet (detailing anonymity, right to withdraw and lack of any impact on their assessment or progression) and were asked to provide signed consent.

Each of the six interviewers interviewed two or three students individually. Before interviewing, all interviewers met to receive any necessary training in qualitative interviewing, review the questions and to agree on an approach to the interviews. The research group again used a semi‐structured interview technique, using the questions as a guide and using prompts and follow‐up questions to explore key topics further.

All interviews were carried out one‐to‐one (interviewer and student) on Zoom, using the auto‐transcribe function to create transcripts. Interviews lasted between 30 and 60 min. The transcripts were checked for accuracy by the interviewers and edited accordingly.

Three members of the research team carried out an initial analysis of five of the interviews to formulate a draft framework. Each member independently identified key themes which were then discussed during a meeting to form this draft framework. Detailed analysis of all interviews (including the initial five) was then carried out by five of the interviewers, using this draft framework and NVivo software. Each transcript was also coded by the stage of the student. Each of the 15 interviews was independently coded by two members of the research team. Following coding, the research team met again, to discuss the findings and to refine and negotiate the draft framework.

## Results and Discussion

**Fig. 2:**
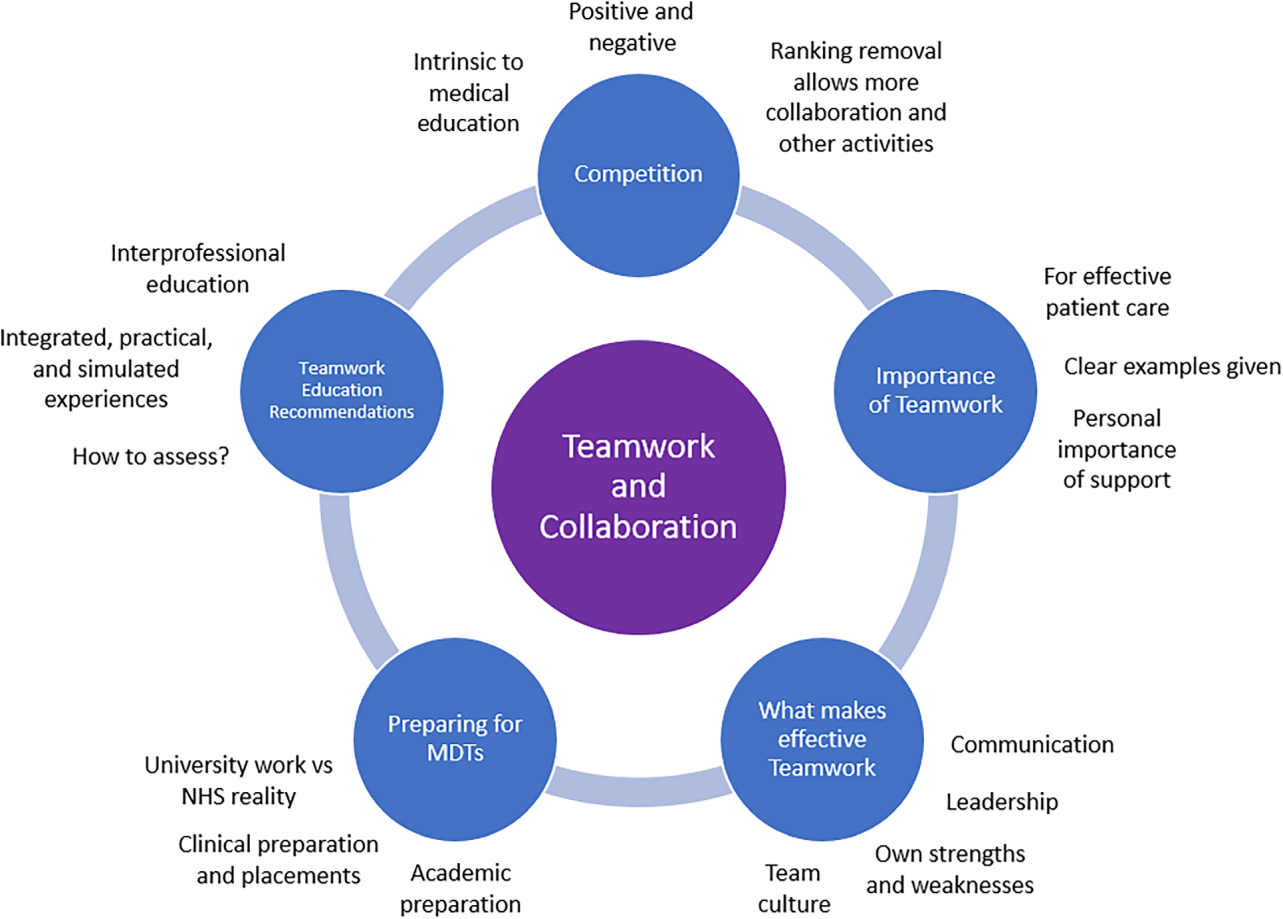
Key themes from interview analysis. The key themes that emerged from our thematic analysis of the student interviews are shown in blue circles. The important sub‐themes are shown adjacent to each theme.

The key themes that emerged from thematic analysis are represented in Fig. 2. These themes were generated from discussion of the draft framework, discussed above. Where quotes are used in this section, they are in italics and labelled with the stage of the student. No other identifying characteristics are reported.

### Competition between students

The concept of competition between students inhibiting teamwork and collaboration was a key finding in our previous paper [3], and we looked to explore this further in this study, especially in the context of changes to rankings, a move to removing norm referencing, and changes to our teamwork education.

#### Positive and negative

Overall, across the interviews, there was an acknowledgement that competition was present, but students did not generally perceive it to be only positive or negative. Most students felt that some aspect of competition was positive and that it might encourage students to try harder and do better. But they also noted, sometimes emphatically, that it could also be detrimental to students' well‐being and morale, including by reducing collaboration. The word ‘toxic’ was used by several students to describe negative aspects of competition, yet many of the same students felt that competition would encourage them to aim for excellence. Please note, whilst quotes given here may be entirely positive or negative in sentiment, over the course of the interview, individual students almost always noted both positive and negative aspects of competition.

Stage 1: *‘I think that's sometimes good, because if there is a competition or ranking you constantly working to improve yourself. But now you're more chill. I'm not saying that you don't study at all, but you're more chill in terms of ranking and sometimes it's, it's hard to make progress, but with competition you are constantly working to improve yourself. To improve your quality of studying, to, to improve your approaches. And this can make you [a] better doctor in the future.’*

Stage 4: *‘Okay. I think with competition, it's different because I love it and I hate it at the same time. It's, it's a driving factor to study. But we're not meant to be super competitive where everyone sits on their own and studies on their own.’*

#### Intrinsic to medical education

Many students mentioned the idea that competition was intrinsic to medical education, and although we no longer actively promote a ranking system, it would still be perpetuated by the students themselves, even if this is detrimental to collaboration between students.

Foundation: *‘there's always competitiveness even if you don't get told, if you find out your marks … even though it's not published if you can find out like oh, I got 10 marks or like 15 compared to someone who got five. There's always going to be that type of competitiveness. But I think if it was lower it would increase the teamwork because people would be more willing to share resources and work together.’*

Stage 5: *‘medical students are already high achievers and they already have, probably have an element of perfectionism in them, so I think that competition is not necessary. And in medical school, competition can easily sway into something being not useful and quite harmful mentally and for their knowledge growth as well.’*

Stage 5: *‘we're all med students, we're all high achieving med students … So at the back of your mind, you are thinking, yeah, these are my friends; these are my colleagues, but they're also my competition.’*

#### Removal of rankings and collaboration

Some students noted that by removing rankings, their focus changed from competition between students, to competition by students working together to do the best they could within the system, increasing collaboration.

Stage 1: *‘it is like a unifying feeling that we're all in this together. Like yeah, like, everyone's still trying to do that, the best they can. So let's support each other in that. So I don't know how to summarize that into feelings per se, but it's a more comforting experience, at least reassuring, I guess … It's us versus the medical school … So that is a more healthy mentality to have to be like, “Okay, let's work together in order to get that grade.”’*

Stage 1: *‘in my opinion like it, it gets rid of the toxicity that would have been the like, the, the competitive mentality … It, it encourages a more collaborative… And you know, a mutualistic approach to be like, “okay, let's work together to pass medical school.” Not: “Oh, I want to be 5 ranks ahead of this person” for example.’*

Stage 2: *‘And so I think that it's better now without the rankings … I worked with two peers and we'd meet up like a couple of times a week to practise for a few hours. And there was never any like hint of, like, oh, well, I'm going to try and do my exam better than you because I want to show you that I'm better or I'm going to be better than you. It was just like it came from the genuine place of like, we all wanted each other to succeed. Like we messaged each other's before the exams to be like, good luck everyone.’*

Some students suggested that by removing rankings, we would allow students to focus on other things that might be just as important to being a good doctor as high grades in examinations.

Stage 4: *‘what people also doing is taking a step back from medicine, but increasing their social development so they can show when their applying for these fields, look, here's why they're in medical school. Here's how many papers I've published, or here's how many activities I've been involved in. I think this has been a really good thing in the sense that it's given people an opportunity to better themselves in other areas.’*

Our students are clearly of the opinion that competition is neither purely good or bad. Most of those interviewed cited both positive and negative aspects. Others have noted the potential for competitive environments to negatively impact medical students [29]. Furthermore, competition seems innate in the medical school environment. Our students indicated that even if our assessments didn't promote competition, they would still compete with each other to some extent. More positive, was the feeling amongst our students that removal of direct competition through ranking was encouraging students to work more collaboratively, to help each other study and that it also allows them to take part in other academic pursuits like research.

### Importance of teamwork

All students agreed that teamwork was important in healthcare, and many mentioned the importance of teamwork in their lives generally, including in their current programme of study.

#### For effective patient care

Students at all stages agreed that good teamwork was vital for effective patient care. This was stated often. Sometimes students gave specific examples, but not always. They know it is important, but do not always articulate exactly why.

Stage 3: *‘So professionally, I think, as a medical student and a future doctor, I think teamwork is important in the sense that you need to work as a team to take care of the patient … So you have your nurses, you have your other fellow doctors, other health care professionals that you have to kind of work together. So for the overall benefit of your patient. Also, I think it's important in terms of communication as well. So that you are communicating and you are working together to make a decision.’*

#### Examples of the importance of teamwork

Students used both positive and negative examples to demonstrate the importance of teamwork in healthcare. In terms of understanding the importance of teamwork, more students gave clear and tangible examples than in our previous study where students knew teamwork was important, but may have lacked the insight to be able to explain *why* [3].

Stage 4: *‘when I was on ED placement … we always get told about teamwork, but actually seeing it in practice is very different, and it makes a big difference to the patient's outcome. And, for example, one person would take bloods, another person would like do the oxygen saturation and and give that person fluids, another person is in charge of talking to the family and making sure the family is aware of what's going on actually saves a lot of time, and it was better for the patient outcome and for the survival of the patient.’*

#### Teamwork and collaboration providing support for students

The personal importance of teamwork to our students was raised, often blurring the boundaries between teamwork in a professional sense, collaboration and working in groups of friends or peers. Regardless, it was generally seen as a supportive element of medical education, fostering inclusion and enjoyment. There was also recognition, especially by more senior students, that it is not necessary to be friends with everyone in a team, but that team members should all be included and demonstrate professionalism.

Stage 1: *‘I think teamwork is quite an important part … because for me, I learn best through discussion. So, like sharing ideas and bouncing it off other people. I think it really benefits me, and I feel like I can do a lot in much less time. And it's far less, you know, mentally taxing even than say, sitting down and trying to read off a textbook, and I think it's more active as well … So in my recent exam, I was able to work in a little bit of a group setting, and it was sort of, like, we motivated each other to keep working to keep sharing ideas.’*

Stage 4: *‘Everyone has social groups, right? So it's very common in a cohort of now nearly 180 people that you will have people that you don't get along with, and that's absolutely normal and fine. But that shouldn't form a bias when you're working together in a team, and I know this will be less so in professional practice. But at the minute it's just making sure that you have a good professional set of values which also incorporates, understanding other people's values as well in it. Understanding people's values is really important. So you don't let it demoralise them or cause a conflict.’*

Our students could clearly appreciate the importance of teamwork with respect to patient care, and some gave clear examples from their placements and other experiences. What also came out in this theme though was the real personal importance of support and collaboration, especially from students in early years. This wasn't such a strong theme in our previous paper [3], perhaps indicating that students are more open to helping each other out, even if it means risking friends getting higher marks in exams. Students in later years tended to refer more to professional teams rather than groups of peers or friends, perhaps reflecting a growth in their understanding and experience. A possible nuance of our interview approach is that we asked about the importance of teamwork as the first question. It might be that students were extremely positive about this because they felt they needed to say the *right* thing to us. It would be interesting in future studies to ask this question later in the discussion to see whether their responses were any different [30].

### Elements of effective teamwork

A small number of key characteristics emerged when we asked students what they thought made good teamwork, as well as what they thought their own strengths and weaknesses were.

#### Communication

Communication was often the first element that students talked about.

Stage 1: *‘I feel the most important thing is communication. It is… It's so vital because… how do you play to each other's strengths if you are not able to communicate? … communication would be the most important asset to have as a team, because through communication as well, you can make sure everyone feels integrated and valued in the team … Cause, when things go wrong, inevitably things will go wrong. It's important to communicate, like communicate and highlight expectations, perhaps ways you can, you know, overcome such barriers in the future.’*

Stage 3: *‘I used to work in a Thai restaurant and I was, you know, my colleagues had like a different language. So they were talking in different language, and I was the only one … who … couldn't understand what they said, and they really didn't include me. So I felt quite like separated, and I realised the importance of like communication as well.’*

#### Leadership

Leadership was often mentioned by students as being important to good teamwork. They also picked up on the need for team members to have clear roles, and that the team leader should be able to identify which team members should be assigned which roles.

Foundation: *‘Because maybe it sounds bad, but I think there are certain people who are best put in a leadership position and there are people who are best put in more of a following position. And that's not to say that anyone is better or worse, because you need both of them to have a team but I think there are certain roles and certain people fit them better than others … But you can kind of tell who is going to do what and you can and in my head, I'm just thinking, maybe we need like an extra person in here who can like placate this guy, or maybe me or someone else should go into a different group because we're gonna butt heads and it doesn't fit. And I get it's like balancing it and thinking that you should work in a team no matter what. But I think if the question is kind of like creating an ideal team, I think it takes effort in finding the right people.’*

Stage 4: *‘A leader is a team player first, but the only difference is, they have the ability to have the perception of understanding the strengths and weaknesses of the other people, making sure that they delegate tasks … as well as facilitating the whole, the team project.’*

#### Team culture

Students often noted the importance of creating a supportive culture within a team, including valuing team members, sharing feedback and working towards a shared goal.

Foundation: *‘If you have a group of people around you that you can trust. It makes everything a lot more easier. Because you can rely on them. They can rely on you, and you get feedback, which is very valuable.’*

Stage 2: *‘When people feel like it's their choice about what they're doing? And some of it was their idea? They're going to be a lot more motivated to do it and with much better ability, I think.’*

Stage 5: *‘I think another important thing actually is having an open culture and a supportive culture where you can, where you don't feel penalised raising ideas.’*

#### Strengths and weaknesses

When asked about their own strengths and weaknesses in teams, students often again mentioned communication and leadership, as well as defining clear goals, giving feedback and being well‐organised. Sometimes these strengths and weaknesses were mentioned without evidence or feedback to support them, but often students gave specific examples to illustrate their answers. Students showed different levels of self‐awareness, not always correlating with how far they were through the programme. Some students also demonstrated some more in‐depth reflection, using their own strengths and weaknesses to gain insight into effective teamworking. Our previous study showed some insight into skill development during the progress of a module [3], but the students interviewed this time more often gave more thorough answers to this question, demonstrating that across the programme, these students had some good insight into their own teamwork development and how this impacts their work in academic and clinical teams.

Stage 1: *‘I feel like I'm able to delegate tasks well. Because I feel like I can take the time to understand what a person's strengths are … if you take the opportunity to be like, oh, “What, what is it that you like?” “What is it that you like? X, Y and Z?” and then work on that. Sometimes it's not the most feasible thing. Sometimes you just have to sit down and say, “can someone do this, this and this?” right, and then sometimes it can almost be like a dictatorial position where you're saying, “do this, this and this”’*

Stage 2: *‘giving like constructive feedback and critical feedback I find really difficult. Like I find it hard to say negative things to people because maybe because I have a tendency to take things on board … I don't want to make them feel like they've done something wrong.’*

Stage 5: *‘I'm still working on kind of learning to step back and let other people. Be like, share their ideas and stuff. And sometimes I can just like jump straight in there and that's not always like the best thing to do. Actually, it's better to just sit back and. Come in a bit later. Yeah, I do that in small groups a lot, where we're just someone shares something interesting and then if I have like a thought or an idea, I'll jump straight in there and share it. But I think I'll just, that's something that I want to work on is being better at. Letting other people share their ideas first.’*

Communication, leadership and culture were all key themes that emerged when students were asked about effective teamwork, including when they talked about their own skill sets. This is reassuring, as clearly these are all important components of teamwork [31, 32, 33, 34, 35] and are concepts that we regularly discuss in our teamwork teaching. Students also provided some very coherent and in‐depth analysis of their own teamwork competence, including illustrative examples.

### Teamwork development: preparing for work and real‐life experiences of the NHS

During our interviews, analysis and discussion of our framework, a key theme was students noticing a difference between how we teach them teamwork in the academic setting (and how their teams work inside the University environment) and how teams actually function (or not) in the real‐world of the NHS. A number of students told us about teams they had observed on placement who they did not feel entirely embodied the ‘ideal’ version of teamwork they might have seen within the academic environment. Both academic and practical examples of teamwork development were referred to.

#### The academic environment

The teamwork examples given from the academic environment were generally positive, demonstrating how they have helped students develop a strong foundation in teamwork in a safe environment. Students often also appreciated why learning these various elements of teamwork was important to their future careers, and often gave examples of how learning from academic teams was transferable to their clinical careers, without being prompted.

Stage 1: *‘And I learned that feedback is an important element. because even in EBL, for example, we give out like feedback on a regular basis … previously I didn't know much about feedback, but but as I enter med school, I know that … sometimes we might have a weakness, and we need to be open to feedbacks and feedback's a really important element.’*

Stage 1: *‘I think a great thing that we're doing is learning from each other's mistakes … learning from each other's insights … EBL is the premise of like, sharing each other's ideas and building on knowledge like that, and asking the right questions. It's sort of to mirror the MDT, right? So in that regard, it's, it's help, it's making me think, oh, this is like stimulating what it might be like in the future. And a lot of my friends have said like, oh, when they went placement as well, they saw, you know, different doctors or different healthcare professionals, nurses, all like, sharing ideas.’*

Stage 2: *‘Like I'm in a, we're doing our DaE at the moment and with that it's been like really helpful in like teamwork in that sense because it's kind of like an going project. And so managing that and understanding how that works in terms of like if I don't meet my deadline, then it's going to impact someone else.’*

#### The clinical environment

The examples students gave of teamwork from the clinical environment were more varied. Some saw, and were part of, positive and well‐functioning teams. Others were able to see where teams had shortcomings, and some seemed a little upset or perhaps even disillusioned by what they were seeing in the real‐world. In both cases, students seemed able to apply their learning about teamwork in the NHS context. This sometimes gave students real clarity, almost a light‐bulb moment, seeing why teamwork education is fundamental to medical training.

Stage 3: *‘So I've seen like resuscitations. And like, I've seen people, people's lives being saved and teamwork being a very important factor. That led to them being saved by the team, and I've seen how like MDT. Like work together to deliver the best practice, like you know, in in in the best interest of the patient.’*

Stage 5: *‘I've understood the importance of like, knowing every everyone's role within a team. I think, I guess I didn't have much exposure to that, because in years one and two, we would be kind of working in, you know, with other medical students and other colleagues in our year for groups and projects. So I think coming into the placement environment has made you realize that the importance of knowing each person's role because that kind of affects their perspective on the situation. And also it shows you what they bring into, they bring to the table in terms of skills and abilities.’*

Foundation: *‘But I think if you looked at the communication of this whole group, from the outside and your job was just to look at how people interact, you kind of think maybe there's a bit of a problem here … Because like people will talk bluntly, or they'll make certain remarks or jokes, and everyone accepts it and I don't know if that's just the culture and that's great and no one has a problem. But for me, not being in that environment. And seeing it from the outside. I was a bit… I didn't find it great. But, again, if it works, and everybody's happy, then it's not a problem.’*

Stage 1: *‘Here's the thing right, with the NHS like, and a lot of other professional bodies, it is quite a competitive environment. So maybe medical school is a soft landing to that … But I hope you can understand like that, for me, at least, my perspective is that the professional world is very, looks very scary and cutthroat. Our medical school is, you know, is promoting … a more teamwork sort of mentality. For, for the benefit, you know it works well. But at the same time it might not, perfectly mirror what that professional world looks like.’*

The comments given by students about examples of teamwork in academia and the NHS are interesting. The academic examples were overwhelmingly positive, and more positive than in the previous study, indicating a move away from the resistance to teamwork we saw last time [3]. It is possible that the reduction in direct competition between students may have increased their sense of psychological safety, allowing them to contribute more easily to group and team activities [21]. The mixture of positive and negative examples of teamwork observed in the NHS demonstrates a good understanding of functional teams, that wasn't as apparent in our previous study. What students seem able to do this time is articulate not only that teamwork is vital, but also why. They also refer to their own personal development here, reflecting on how their own skills have improved and how this is preparing them for being doctors.

### Recommendations for enhancing teamwork in medical education and higher education

During analysis and discussion of our results, we felt that several ideas raised by students might be useful for future teamwork education development, in healthcare and higher education more broadly.

#### Interprofessional education

Interprofessional education was not mentioned specifically in the questions we asked, but a number of students raised it as something they wished they had more of, noting how valuable they felt it was to developing their teamwork for a career in healthcare. There was a feeling of positivity about working with other healthcare professionals and a real sense of inclusion and wanting to work together for the good of the patients. This was not a concept that emerged clearly in our previous paper [3] so perhaps the increase in visibility of IPE both in our programmes and across the Faculty, meant students were more aware of it, and this made them want more of it.

Stage 1: *‘Time for dementia is really really good where we do it with other, with other students … it'd be nice to get more like and of communication between the different healthcare professionals … say … you did a joint situation with the paramedics, and then you went and like had a chat with some midwives, and this I'd I'd go and do it like voluntarily … I'd be really interested to do that.’*

Stage 2: *‘I'd hate to be surrounded by just medics. I don't think… I don't think that's good for anyone … I jump at the chance to be put with other healthcare professions other than doctors, because I think you often learn a lot more um in those placements than just following around a consultant … I would like some more interdisciplinary work. We did, we had EBL last week with some physiotherapists, and it was really good.’*

#### Integrated, practical and simulated experiences

Students also noted how important different teaching modalities are in delivering effective teamwork training, including how important clinical simulations and structured feedback are to them.

Stage 2: *‘I feel like lectures on teamwork aren't always the best way to teach teamwork. It's like, you know, they have that question where it's like, can you teach empathy? … I'd say maybe just maybe having more like a focused, like smaller focused group on how or maybe more structured feedback, I'd say that could be a good thing.’*

Stage 5: *‘I think having more of the simulation in clinical skills. They are really, really vital and useful, and integration of not just medical students but also different healthcare professionals coming in; so, like, radiography students and different people, and it makes me think about, you know, simple things that have a big impact, like, “are radiography students allowed to put a cannula in?” That really helps especially in the simulation and emergency care environment.’*

#### How to assess teamwork

Finally in terms of developmental ideas that emerged, students seemed undecided about whether assessment of teamwork during their programme was helpful or not. They recognised the need for accountability in a team and that without this, some of their peers might not contribute as much as they should. But they also noted that assessments can be restrictive and may inhibit creativity. There was also the implication that some marking criteria may not be fully aligned with the overarching goals of the programme and seem artificial or fake.

Foundation: *‘Because assessment is necessary to assess obviously, and also it can be a good motivator for certain people. However, if you're talking about creative work or collaborative work … Taking away an assessment can benefit you because you have more freedom, you have more control of what you're doing. And then you don't have to worry too much about being marked on things rather than what is your goal for this task, and can you achieve it?’*

Stage 3: *‘I think the assessment part needs to be removed. But then, if you remove the assessment, no one participates.’*

Stage 5: *‘I think, generally, in terms of teamwork skills we've been taught, we've been encouraged to develop them since year one … But I think I guess one thing to mention is that, it I think, it's difficult when you're being assessed in those situations as well. It can make kind of your perception of teamwork a little bit skewed and biased. Because when you're being assessed, you're going to say things and do things, when you're working with the team, for your tutor to mark down to mark like, you know, to give you a score in the mark scheme.’*

It was interesting that our students noted the importance and value of IPE here, in line with the broad consensus in healthcare education [36]. The increased visibility of IPE might account for that, but perhaps the general shift to increased collaboration means they are more willing to work with other healthcare professionals in general. They also acknowledged the need for teamwork education to be at least in part more practical, again in line with the literature which suggests teaching transferable skills in authentic, workplace‐like environments [10]. The positive and negative aspects of assessing teamwork were picked up on here. This may reflect the fact that we, in line with many others in higher education, are only part‐way along our journey to establish robust skills assessments [37].

### Limitations

Whilst this study has obtained in‐depth data on students' perceptions of their experiences, we acknowledge the limitations of our study design. Because engagement with the interviews was entirely voluntary, it is likely that the students who came forward were already interested in teamwork, or medical education, thus biasing our sample somewhat. Students who attended the interviews were rewarded with a £20 voucher to recognise the time they had given up, but we do not expect this to counteract the likely bias in our sample. Our sample size is also very small, but we were interested in obtaining detailed perspectives, not expecting to gain an exhaustive list of every possible experience our students have had relating to teamwork. We do acknowledge that the low numbers of students we interviewed from each cohort meant we could not effectively carry out a vertical analysis comparing experiences of different year groups. We would like to continue to investigate students' perceptions of teamwork through higher education and intend to pursue a mixed methods approach, allowing greater sample sizes whilst maintaining the richness of the qualitative data we have presented here.

## Conclusions

This paper reports findings from an in‐depth qualitative study of students at all stages of an undergraduate UK medical degree. We note our relatively small sample size, but the data we have acquired is rich and detailed, giving real insight into students' experiences and perceptions. As described, whilst some of the more nuanced findings relate to the UK medical training process, much of what we conclude can be applied across higher education, regardless of discipline.

This study demonstrates a shift in our students' attitudes to competition and collaboration since our previous study, likely to be due to a number of factors including our revised curriculum, removal of rankings for graduate Foundation placement allocation and a move away from norm referencing. Several students described working with others rather than against them, to achieve their best together. Perhaps this represents more collaboration within groups but competition between groups, or even between students and the School.

Students demonstrated deeper insight into teamwork this time, explaining examples of experiences of teams in the academic and clinical environments. They appreciated not only that teamwork was important, but related it to their own experiences and development in a clear and tangible way. They also reflected on their own strengths and weaknesses and in some cases showed real clarity on where they needed to develop their own skills. We attribute this, at least in part, to our enhanced teamwork curriculum, and we recommend an evidence‐based approach to transferable skills education, just as we do with subject‐specific content.

Teaching teamwork continues to be a challenge, not least how we allow individual and organic development of these skills whilst assessing students' progress. Based on data from these interviews, applied and authentic activities are favoured by students to enable them to develop teamwork skills, such as simulations, group projects and IPE. We also need to continue to improve assessment of teamwork, ensuring that we are testing them on working and achieving something as a team. Removal of unnecessary competition and comparison between students is recommended too, in favour of a culture where we promote collaboration and encourage students to work together to achieve their best.

## Conflict of interest

The authors declare no conflict of interest.

### Peer review

The peer review history for this article is available at https://www.webofscience.com/api/gateway/wos/peer‐review/10.1002/2211‐5463.13915.

## Author contributions

HRW and PM designed the project, based on a previous study by HRW *et al*. All authors carried out interviews with students and prepared transcripts. HRW, PM and DZ did an initial analysis of the interview transcripts. HRW, PM, JC, HS and DZ did an in‐depth analysis of all of the interview transcripts. HRW led the research team and prepared the manuscript, with input from all authors.

## Data Availability

The data that support the findings of this study are available from the corresponding author (helen.watson@plymouth.ac.uk) upon reasonable request.
